# Impact of late and prolonged working life on subjective health: the Swedish experience

**DOI:** 10.1007/s10198-018-1005-z

**Published:** 2018-09-06

**Authors:** Dominique Anxo, Thomas Ericson, Chizheng Miao

**Affiliations:** 1 Department of Economics and Statistics, School of Business and Economics, Linnaeus University, 351 95 Växjö, Sweden; 2 Linnaeus University Centre for Discrimination and Integration Studies, Linnaeus University, Växjö, Sweden

**Keywords:** Extending working life, Self-assessed health, Retirement, Sweden, I12, J14, J26

## Abstract

This paper explores the relationship between the prolongation of working life and subjective health. Drawing on a unique combination of longitudinal data and the results of a postal survey in Sweden, we investigate the health consequences of extending working life beyond the normal retirement age of 65. To do this, we compare the health status of two groups of retired people: one group who left the labour market completely at the age of 65, and a second group who remained in employment after the age of 65. Using a standard linear probability model and controlling for a range of socio-economic variables as well as previous labour market experiences, perceived life expectancy, pre-retirement income and health, our estimations show that those continuing to work after 65 on average display a 6.8% higher probability of reporting better health during retirement than those leaving at the age of 65. However, we find that this positive correlation between the extension of working life and health is only transitory. After 6 years of retirement, the health advantage of working after the normal retirement age disappears. Furthermore, we did not find any evidence that working after the age of 65 is positively correlated with physical fitness, self-reported depressive symptoms or well-being.

## Introduction

Like many post-industrial societies, Sweden has over time experienced a long-term tendency towards a shortening of working life due to inter alia longer education and earlier exit from the labour force. Combined with an ageing population, this tendency has created serious challenges for the long-term financial sustainability of the Swedish social protection system. One policy measure to address this issue was to encourage workers to voluntarily delay their retirement and to continue working beyond the standard age of retirement.

A comprehensive public pension reform was implemented in 1999 and the Swedish pension system is nowadays based on lifetime earnings and a flexible retirement age. There are three components in the Swedish public pension system: income pension, based on a person’s full lifetime earnings and financed on a pay-as-you-go basis; premium pension, which is a smaller and funded part of the income-based pension; and guarantee pension which provides a basic income for all persons, irrespective of their previous lifetime earnings. The statutory pension system is complemented by collectively bargained occupational pension schemes, which cover about 90% of the workforce. The pension system provides the freedom to combine work with retirement, and both employees and self-employed persons are entitled to pension benefits. Statutory income pension is payable from the age of 61 for both men and women. However, the size of the pension will increase the longer the retirement is postponed, and the financial incentives for postponing retirement are strong. Nevertheless, the standard age of retirement in Sweden is 65 years for both men and women [2].

If longer working lives may improve the financial stability of the pension system, there could also be increased costs, for example, for healthcare should the prolongation of working life lead to a deterioration of health. On the other hand, paid work activities and social relations associated with employment might also lead to long-term positive effects for health and well-being also for people over the standard age of retirement. The relatively high employment rate of Swedish older workers^1^ in combination with the possibility to voluntarily extend working life beyond the former statutory age of retirement at 65 makes Sweden an interesting case study with respect to the health consequences of late retirement and prolonged working life.

Drawing on a unique combination of longitudinal data and a postal survey sent to 20,000 elderly citizens aged 65 or older living in Sweden, the main objective of this paper is to explore the extent to which a prolongation of working life has an impact on health. Senior employment (i.e., employment over the age of 65) may affect individual health in two opposing directions: On one hand, prolonging work beyond the standard retirement age may lead to a deterioration in health. Previous studies have shown that retirement is beneficial to health through different mechanisms such as the relief of work-related stress and the increasing time on physical exercises [10, 12, 15, 24]. On the other hand, literature from epidemiology and health economics has shown that social relations are strongly positively correlated with health [4, 25]. Snyder and Evans [27] argue that social interactions associated with senior employment might lead to health benefits. If this is found to be the case, then senior employment would benefit society and should be encouraged further by policy makers.

Focusing on current retirees, we investigate whether working beyond the standard Swedish retirement age of 65 has any impact on the current health of older people. Specifically, we compare the current health status between the current retirees who had worked after age of 65 with those who had never worked after age of 65. One advantage of focusing on the retirees as a group is that we can assess the short- and long-term health effects of prolonging working life after 65, since those who recently have withdrawn from the labour market might have more social connections than others. In this paper, we define current retirees as the treatment group if an individual has worked beyond the standard retirement age of 65 for at least 6 months. The control group comprises individuals who definitively exited the labour market and retired at the standard retirement age of 65. To access the health impact of working after age of 65, we use a subjective self-assessed health measure from the postal survey as the main outcome variable. Specifically, we asked respondents to assess their current health condition using a five-level Likert scale ranging from ‘very bad’ to ‘excellent’. Despite criticism of subjective measures, self-assessed health reporting is one of the most commonly used health measures in empirical health economics and has been shown to be a good predictor of objective health status [8, 22, 28].

One major empirical challenge in estimating the health effects of senior employment is related to potential selection effects. People who chose to work beyond the standard retirement age may belong to a healthier group of people, which may lead us to spuriously conclude that senior employment has a stronger positive health effect than is actually the case (selection effect). To minimize any estimation bias, we use the total number of sick leave days between age 59 and 64 from Swedish register data to proxy the individual’s health condition before age of 65.^2^ Previous Swedish empirical evidence has shown that there is a clear association between sickness leave data and future all-cause and cause-specific mortality [6]. However, a limitation of this study is that we cannot claim the results as causal effects due to other unobserved cofounding factors that could have an influence on health.

This paper contributes to the previous literature in two central ways: first, previous research has studied the health effects of early retirement in comparison to working until the standard age of retirement, while our study focuses on the health effects of working after the standard age of retirement. Given the increasing employment rate of senior workers, we think it is highly relevant to study the health effects of prolonging working life after the standard age of retirement. Second, we study the health consequences of prolonging working life using a national representative sample which underpins the external validity of our results. In contrast, previous studies have used different identification strategies where the interpretation of the results has been limited to specific gender and occupational groups.

Using a standard linear probability model, we control for a range of socio-economic variables, previous labour market experiences, previous income, perceived life expectancy and previous health. Our estimations show that the treatment group on average displays a 6.8% higher probability of reporting better health during retirement than the control group. However, we find that the positive effect on health of extending working life is only transitory and mainly concerns medium-skilled workers. After 6 years of retirement, the health advantage of having worked beyond the standard retirement age vanishes. Furthermore, we did not find any evidence that working after 65 would improve physical fitness, well-being or decrease the likelihood of self-reported symptoms of depression among the retirees.

Our knowledge about the health effects of active ageing and retirement and how previous studies have dealt with the methodological problems are discussed in “Literature review”. A description of the data, some methodological considerations and descriptive statistics are provided in “Methodological considerations and descriptive statistics”. The baseline results of our estimations are described in “Baseline results”. In “Heterogeneity”, we analyse whether health outcomes vary according to sub-groups (gender, marital status, labour income and skill level). We also investigate the effect on other health-related outcomes, followed by some concluding remarks in “Conclusion”.

## Literature review

It has been discussed and analysed for a long time whether retirement harms or improves health (see Minkler, [23] for an early review). One argument is that retirement itself is a stressful life event, and may lead to losing friends and supportive networks. Retirement may also be accompanied by feelings of loneliness and with a focus on the negative impact of age [7]. On the other hand, retirement can be a rewarding change of lifestyle that helps to improve health by removing work-related stress and other possible physical or mental burdens associated with paid work [16].

There are basically two alternative empirical strategies to study the health effects of retirement: (1) To study the health outcome from an early retirement compared to the health outcome from retirement at an older age; (2) To study the health outcome from a later retirement compared to the health outcome from retirement at a younger age. The former alternative (1) is the most common empirical strategy used in the literature, due to the fact that a majority of the policy reforms have been targeted towards early retirement. The second alternative (2) will probably become more common in the future as many countries recently have implemented policy measures aimed at prolonging working life and postponing workers’ retirement. It should be noted that the health outcome of a later retirement is largely driven by the health outcome of continued work in the old age. Consequently, there is a duality between the health outcome from later retirement and the health outcome from continued work after the standard age of retirement.

Early retirement entails an early exit from the labour force, which may both have positive and negative health effects depending on the worker’s health at the time of retirement as well as the working conditions in the previous job. Recent studies have tried to control for the endogenous effect of retirement on health by controlling for workers’ health status before retirement. The key method is to use exogenous changes of retirement policies which can identify changed retirement behaviour independent of workers’ health status (see Table 1 for an overview). Charles [10] and Neuman [24] used age-specific retirement incentives provided by the US Social Security regulations as instrumental variables. These studies indicate that retirement has a positive effect on subjective measures of health (e.g., self-assessed health, well-being), but not on objectives measures (e.g., limitations in activities of daily living, diagnoses of specific diseases). In a European context, Coe and Zamarro [12] use the Survey of Health, Ageing and Retirement in Europe (SHARE) dataset in a multi-country setting. They use country-specific early and full retirement ages as indicators against which retirement behaviour can be studied. By exploiting the discontinuities in retirement behaviour across countries, they find significant evidence that retirement has a health-preserving effect on overall general health. Interestingly, Heller-Sahlgren [19] also using the SHARE dataset finds that retirement does not have any immediate significant negative effect on mental health, but that there is a significant negative effect in the long term. Several studies have found positive health effects of policy reforms that induce early retirement (Coe and Lindeboom [11], Bloemen et al. [5], Hallberg et al. [18]). However, there are several other studies based on data from various European countries that arrive at contradictory conclusions regarding the health effects of retirement. There is also fairly little evidence on the mechanisms through which retirement affects health. Insler [20] finds that less smoking and more physical exercise seem to improve health after retirement in the US, implying that with more leisure time many retirees practice healthier habits. Eibich [15] shows that retirement in Germany has a significant and positive effect on self-reported health and mental health, and decreases out-patient care utilization. Furthermore, time-use data suggests that health improved due to relief from work-related stress and strain, an increase in sleep duration, and an increase in physical activity.

There are relatively few studies on the health effects of later retirement. Calvo et al. [9] using the HRS panel find that both subjective physical and emotional health are maximized when retirement takes place at the standard age of retirement (62 years) or later. Their result indicates that there is no pronounced health disadvantages associated with late retirement. A recent study explores the health impact of paid work beyond standard retirement age in England. Using three waves of the English Longitudinal Study of Ageing and controlling for baseline socio-economic characteristics as well as work and health history, Di Gessa et al. [14] find no statistically significant health effect of working beyond state pension age. A recent Swedish study by Hagen [17] finds no effects on drug prescription, hospitalizations, and mortality after a reform that increased the age at which female local government workers were entitled to full pension benefits from 63 to 65. On the other hand, Atalay and Barret [3] found that raising the age at which Australian women were entitled to pension benefits from 60 to 65 had a detrimental effect on mental and physical health. Lalive and Staubil [21] found an increasing mortality of Swiss women after an increase of retirement age from 62 to 63 and from 63 to 64. This effect is however small and only statistically significant after the increase of retirement age from 63 to 64.

**Table 1:** Literature overview

Studies	Data	Empirical method	Health effect of retirement
Atalay and Barrett [3]	National Health Surveys, Australia, cross-sections several years	IV; eligibility age reform for women	(+) Positive effect on objective health indicators, no effect on subjective measures
Bloemen et al. [5]	Dutch register data, panel database	IV; early retirement reform	(+) Early retirement decreases mortality
Calvo et al. [9]	Health and Retirement Study (HRS), United States, panel survey	FE, RE, IV; changes of full retirement age and unexpected early retirement windows	(−) Early retirement has a negative effect on subjective health (+) Late retirement has a positive effect on subjective health in the short run
Charles [8]	HRS, United States, panel survey	FE, IV; age retirement incentives, men	(+) Positive effects on subjective well-being
Coe and Lindeboom [11]	HRS, United States, panel survey	IV; early retirement windows, men	(+) Positive effects in the short run, no effects in the long run
Coe and Zamarro [12]	Survey of Health, Ageing, and Retirement in Europe (SHARE), panel survey	IV; age-specific retirement incentives	(+) Positive effect on general subjective health
Di Gessa et al. [14]	English Longitudinal Study of Ageing, panel survey	Longitudinal association between paid work beyond state pension age and health	(+/−) No effect on depression, sleep disturbance and somatic health
Eibich [15]	German Socioeconomic Panel Study (SOEP), Germany, panel survey	RD; based on German pension incentives	(+) Positive effect on self-reported and mental health
Hagen [17]	Longitudinal Database on Education, Income and Employment (LOUISE), Sweden, panel database	DD; raised age entitled to full pension benefits	(+/−) No effect on mortality or health care utilization
Hallberg et al. [18]	Administrative register data Statistics Sweden, panel database	DD; Early retirement scheme of military officers	(+) Reduces mortality and inpatient care
Heller-Sahlgren [19]	SHARE, panel survey	FE-IV RD; age-based discontinuities within state pensions	(−) Negative long-run effect on mental health, no effect in the short run
Insler [20]	HRS, United States, panel survey	IV; self-reported retirement probabilities	(+) Positive effects on a health index based on both subjective and objective sources
Lalive and Staubli [21]	Swiss Social Security data Panel database	RD; increase of full retirement age of women	(+/−) Small decreased mortality
Neuman [24]	HRS, United States, panel survey	IV; age retirement incentives, spouse’s labour supply	(+) Positive effect on subjective health

*DD* differences in difference estimation, *FE* fixed effect models, *IV* instrumental variables estimations, *RE* random effect models, *RD* regression discontinuity design

In this paper, we use an empirical strategy (2) where we compare the health outcome from late retirement with the health outcome from retirement at a younger age. In contrast to previous studies, the late retirement is not induced by a policy change. In our case, the late retirement is based on individuals’ voluntary postponement of retirement, which is increasingly common and partly encouraged in the Swedish pension system. This means that we cannot claim to find any causal relationships between retirement and health. Nevertheless, we think that our study is motivated by its focus on the health outcome of working beyond standard age of retirement, which in the case of Sweden was at the time of our survey 65 for both men and women (as shown by Fig. 4 in the appendix that displays the distribution of the actual age of exit, 65 years appear to be the norm of retirement in Sweden). Individuals that work after age of 65 (hereafter senior workers) may experience both better and worse health compared to individuals leaving the labour market at 65 or earlier. A better health status can be expected in so far that senior workers may hold jobs that enhance their mental and physical health by providing an active life and flexible working hours that are adapted to their capacities and capabilities (in addition to having good health and good working conditions). However, senior workers’ health might deteriorate faster than retired people’s health because retirement in itself has proven to enhance health. It is also possible that many senior workers choose to stay in the labour force after the standard age of retirement as a result of previous health problems and a changed occupation which made it possible or necessary to continue working beyond 65. It is thus necessary to study the health effects of work after the standard age of retirement to fully understand the mechanisms that influences the health effects of retirement.

## Methodological considerations and descriptive statistics

### Data

To identify and compare the health and well-being of senior workers continuing to work after 65 with those who retired at the normal retirement age, we combine one unique postal survey with Swedish register data, the Longitudinal Integration Database for Health Insurance and Labour Market Studies, LISA.^3^ The original survey questionnaire was designed for studying the working life and well-being of elderly people. Between November 2014 and January 2015, Statistics Sweden (SCB) conducted on our behalf a postal survey among 20,000 older people born between 1938 and 1949 (aged 65 and above at the time of the survey). Since SCB did not know people’s labour market status at the time of survey, they sent two types of survey questionnaires, one designed for the survey respondents that were working at the time of survey and another designed for the survey respondents that had withdrawn completely from paid work at the time of the survey. About half of a total of 90 questions were common to the two questionnaires, while the remaining half was specifically addressed to the two groups.^4^

The main objective of the survey was to collect some individual and socio-economic characteristics/factors not directly available from the register database LISA but considered to affect the decision to stay on the labour market after 65 or leave at or before 65. The questionnaire therefore contains questions regarding inter alia some background information of the respondent’s parents, the respondent’s marital status, current and past self-assessed health, work and life history, current and past working conditions, their main motivations to continue or stop working, social relations, personality traits as well as subjective current and life-as-a-whole life satisfaction. A set of socio-economic variables, from the register database LISA was linked to each of the survey respondents. This gives us a rich set of data regarding previous work trajectory, income and life history from 1990 to 2012.

To investigate the health consequences of prolonged working life after the normal retirement age of 65, we focus on current retirees using the sample of respondents that had withdrawn completely from paid work at the time of the survey. One advantage of focusing on this group is that we can examine both the short- and long-term health effect of prolonging working life after 65. Our sample contains 8022 retired individuals. Among them, around 34% retired before 65, 41% retired at 65 and 25% retired after 65. (See also Fig. 4 in the appendix for the distribution of the actual age of exit from the labour market).

In the survey, we asked the respondent to report the age at which he/she definitively left the labour market and the total number of years they have worked after 65. Based on these two questions, we construct our control and treatment groups. The treatment group is defined as those individuals who have worked at least half a year after 65.^5^ The reason for this time restriction is that it is likely that people may continue to work for a few months after they have reached the age of 65 just to finish any existing tasks from the last job. The control group includes retired individuals who definitively exited the labour force at 65 and had never returned to the labour market. Due to the definition of treatment group, we restrict the sample to individuals who are 66 or above at the time of survey. After imposing these age restrictions and definitions of treatment and control groups, the sample reduces to about 5300 observations.

To compare the health outcome of retirees with working experience after 65 with those who left the labour market definitively at the normal retirement age of 65, we use the survey question about the self-assessed health status at the time of survey. The self-assessed health status is measured using a five-point Likert scale: excellent, very good, fair, poor and very poor. Self-assessed general health status is thus our main outcome variable. In addition to self-assessed general health, the survey also includes questions regarding physical fitness, about mental health and life satisfaction. We let these additional variables measure different aspects of health. In the survey, we asked respondents to report physical fitness through the question whether they have difficulty in climbing the stairs to different floors. The same question is asked both at current age and at the age of 64. We create a binary variable of deterioration of physical health, which equals 1 if the individual has experienced a deterioration of physically health between the current age and age 64 and the variable equals 0 otherwise. Moreover, the survey also includes questions regarding mental health (feeling depressed). We create a “depression index” based on four different questions in the survey questionnaire.^6^ The index is scaled between 1 and 5 where 1 indicates the least depressed and 5 indicates the most depressed. Furthermore, respondents also report their current and life-as-a-whole life satisfaction. Those two variables are measured with a 10 scale point from very unsatisfied to very satisfied. After some data cleaning, our final sample includes 4143 observations.

### Empirical strategy

To study the post-retirement health effect of working after normal retirement age, we collapse the ordinal self-assessed health variable into a dichotomous variable. The binary health variable equals to 1 if the self-assessed health is excellent or very good and equals to 0 otherwise.^7^ The empirical model is specified as:

$${y_i}={\beta _0}+{\beta _1}{x_i}+Z{\beta _3}+{\varepsilon _i}.$$

In the model, *y*_*i*_ is the binary health variable for individual *i. x*_*i*_ is the treatment variable, which equals to 1 if individuals had worked after 65 (treatment group) and equals to 0 if they had left the labour market at 65 (control group).

Z is a vector of control variables which may influence health and retirement decision.^8^ We first control for demographic variables like age categories, sex, marital status and immigrant status. In the survey, we also asked whether the respondent’s father and mother died before 65. Perceived life expectancy may also play a role in the retirement decision. If an individual’s parents died before 65, especially due to health reasons, it is likely that her/his retirement decision will also be affected.^9^ To control for factors affecting the decision to prolong working life after 65, we also make use of register data. Previous labour market experience may play an important role in determining work in later life, we therefore control if individuals started to work before age of 20, if they have ever been self-employed, along with the total number of unemployment days between age 59 and 64. Moreover, both health and retirement decisions are likely to be correlated with individual financial conditions before 65. We thus control for both total labour and capital income between age of 59 and 64. Furthermore, to control the selection of healthy people into the treatment group, we include a variable that measures the total number of sickness days between age of 59 and 64. This variable serves as a proxy for individual health status before retirement age. A detailed variable description can be found in Table 6 in the appendix.

To estimate the impact of prolonging working life on subjective health, we use a linear probability model since we are mainly interested in the average effect of the treatment on the outcome. A robustness analysis shows that there is a small difference of the average effect between linear and non-linear models (see Table 8 in the appendix).

### Descriptive statistics

**Fig. 1:**
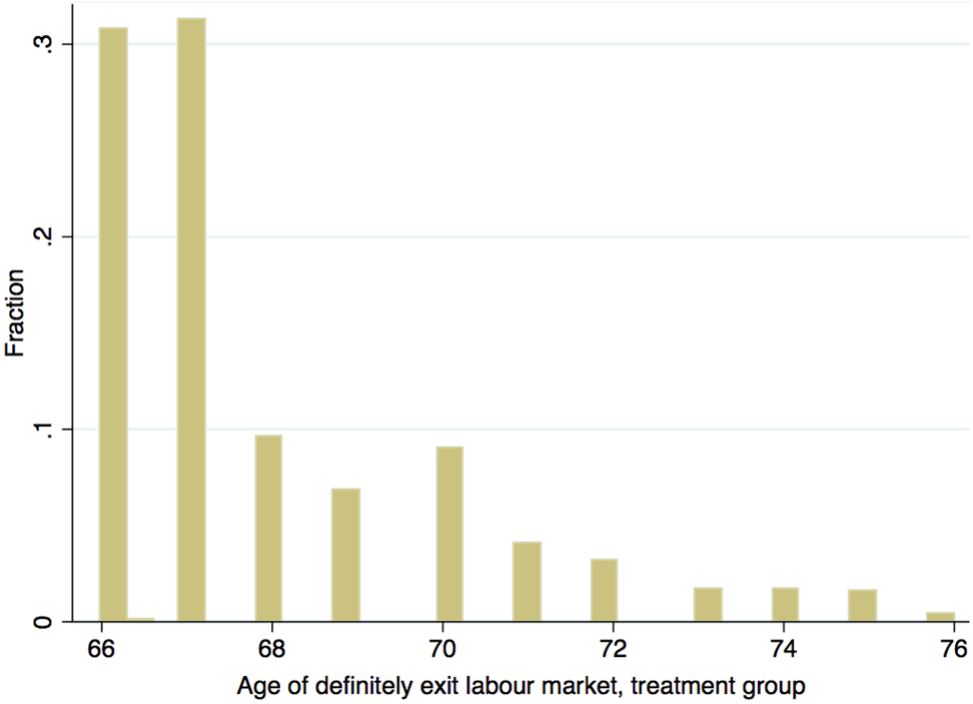
Actual age of exit from the labour market, treatment group

The number of observations in the treatment group (i.e., individuals having worked after 65) and control group (individuals who have never worked after 65) amounts, respectively, to 2177 and 1966 individuals. Figure 1 shows the distribution of age of exit for the treatment group. We find that a majority of individuals retire at age of 66 or 67. This can be partly ascribed to the fact that the prevailing upper limit of employment with statutory employment protection in Sweden is 67-year-old.

**Fig. 2:**
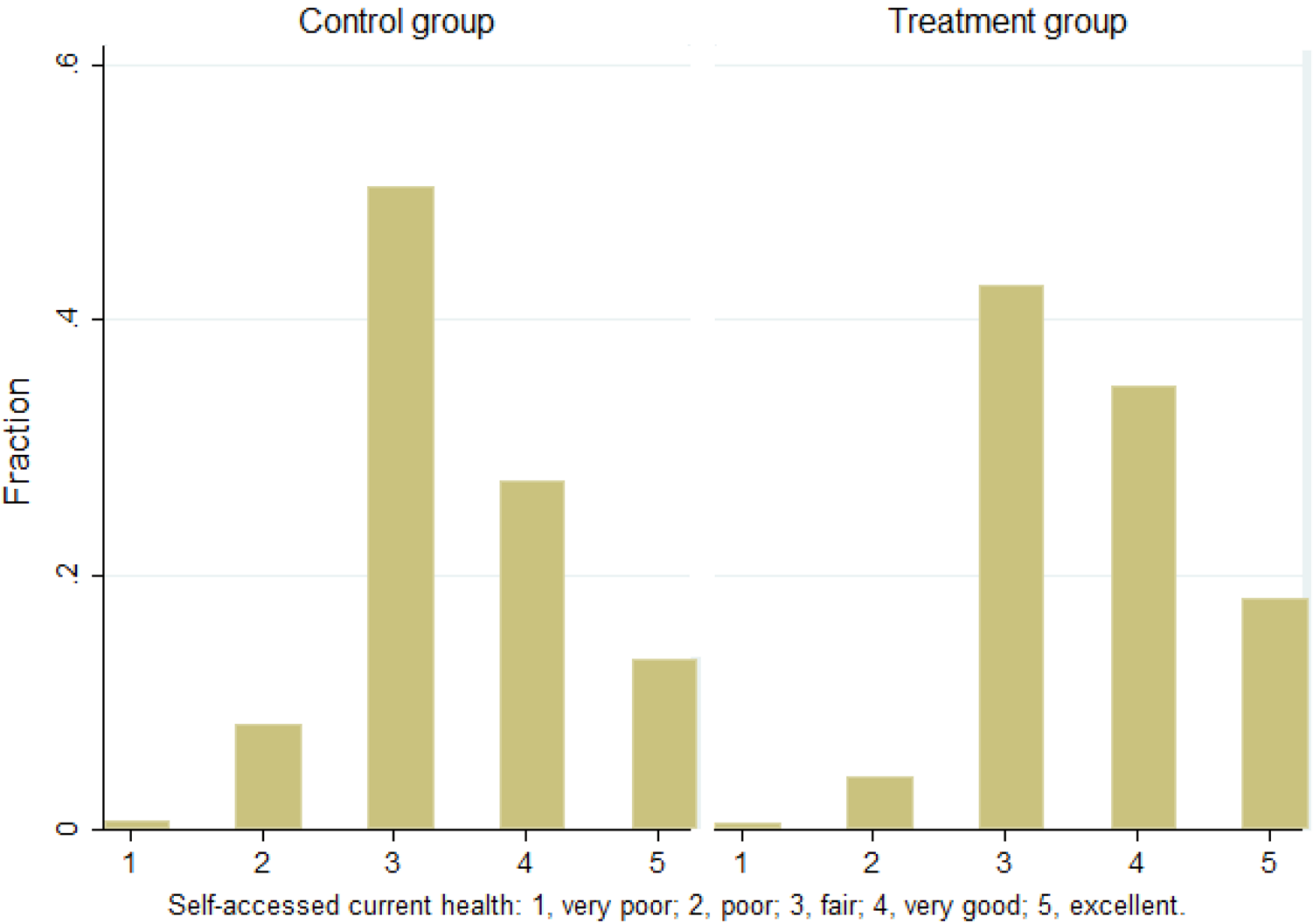
Self-assessed health status, control and treatment groups

**Fig. 3:**
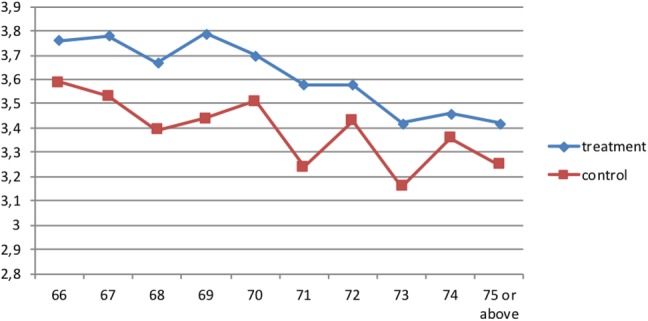
Current mean self-accessed health, by age and treatment status

**Fig. 4:**
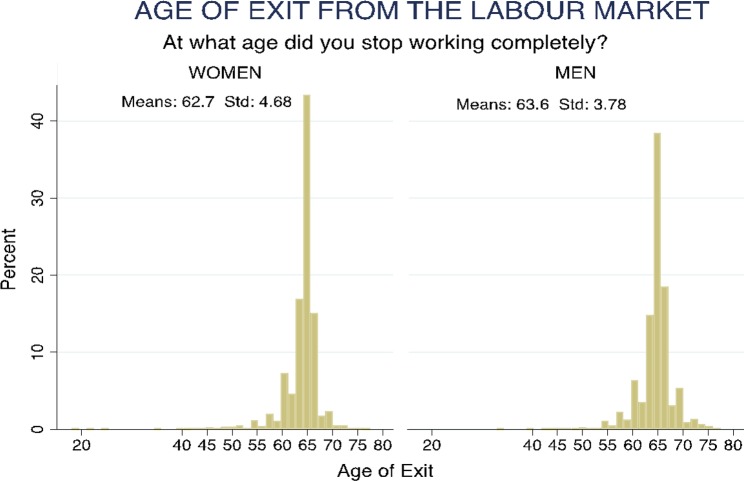
Age of exit from the labour market among retired respondents

Figure 2 displays the distribution of self-assessed health status for both the treatment and the control group. Overall, we see that the median score is 3 (fair) and a larger proportion of people score 4 (very good) and 5 (excellent) among the treatment group compared to the control group. Very few people in both groups report poor or very poor health. Figure 3 shows the mean score of self-assessed health across ages for the two groups. We see that the average health score declines with age for both groups in the sample and the treatment group is on average healthier than the control group at each point of age.

An analysis of descriptive statistics (see Table 2 below and Table 6 in the appendix for the definitions of the variables) confirms that the treatment group is on average healthier than the control group. The mean health difference between the two groups is about 0.22 and it is statistically significant. When we transform the original five Likert self-assessed health to the binary self-assessed health variable, around 53% people in the treatment group report that they have either very good or excellent health compared to about 41% in the control group. Regarding educational attainment, the treatment group has on average a higher education than the control group. This is in line with previous research showing that people with higher education are more likely to delay retirement [2] and have on average a stronger propensity to invest in health. Turning to financial conditions, we find the senior citizens in the treatment group have significantly higher labour and capital income compared with the people in the control group. Regarding the cumulative unemployment spell between 59 and 64, the treatment group has on average 6.7 fewer unemployment days than the control group. Concerning the cumulative number of sickness days between 59 and 64, the difference between the treatment and control group amounts to about 36 days, meaning that the treatment group on a yearly basis is taking on average 6 fewer sickness days than the control group. This illustrates a potential positive selection of healthier people in the treatment group. In other words, healthier people are more likely to delay their exit from the labour market (See also [2]).

**Table 2:** Descriptive statistics. *Source*: Own calculations, based on register data, LISA database from Statistics Sweden and our survey questionnaire

	All	*N*	Treatment	*N*	Control	*N*	Differences
Outcome variables							
Self-assessed health (current)	3.558 (0.846)	4143	3.656 (0.833)	2177	3.446 (0.850)	1966	0.213***
Self-assessed health (binary variable)	0.471 (0.499)	4143	0.528 (0.499)	2177	0.407 (0.491)	1966	0.121***
Physical condition (binary variable)	0.253 (0.435)	4143	0.254 (0.435)	2177	0.252 (0.434)	1966	0.002
Depression index	2.214 (0.695)	3901	2.166 (0.690)	2070	2.267 (0.697)	1831	− 0.101***
Current life satisfaction	7.838 (1.682)	3998	7.889 (1.638)	2122	7.780 (1.729)	1876	0.109**
Life-as-a-whole life satisfaction	8.087 (1.467)	4038	8.118 (1.451)	2132	8.052 (1.484)	1906	0.066
Control variables							
Male	0.551 (0.497)	4143	0.614 (0.487)	2177	0.482 (0.500)	1 966	0.131***
Immigrant	0.0770 (0.267)	4143	0.080 (0.272)	2177	0.073 (0.261)	1966	0.007
Age of 66–67	0.372 (0.483)	4143	0.320 (0.467)	2177	0.429 (0.500)	1966	− 0.109***
Age of 68–69	0.184 (0.388)	4143	0.228 (0.420)	2177	0.135 (0.342)	1966	0.093***
Age of 70–71	0.178 (0.382)	4143	0.195 (0.396)	2177	0.159 (0.366)	1966	0.036***
Age of 72–73	0.131 (0.337)	4143	0.125 (0.331)	2177	0.137 (0.344)	1966	0.012
Age of 74–76	0.135 (0.342)	4143	0.132 (0.338)	2177	0.139 (0.346)	1966	− 0.007
Single	0.108 (0.311)	4143	0.116 (0.321)	2177	0.100 (0.300)	1 966	0.016**
Married	0.764 (0.424)	4143	0.749 (0.434)	2177	0.781 (0.413)	1966	− 0.032**
Divorced	0.064 (0.244)	4143	0.077 (0.266)	2177	0.049 (0.217)	1966	0.028***
Widow	0.063 (0.244)	4143	0.058 (0.266)	2177	0.070 (0.255)	1966	− 0.012
Elementary school at 64	0.220 (0.414)	4143	0.179 (0.383)	2177	0.266 (0.442)	1966	− 0.087***
Secondary education at 64	0.423 (0.494)	4143	0.402 (0.491)	2177	0.446 (0.497)	1 966	− 0.044***
Tertiary education at 64	0.356 (0.479)	4143	0.418 (0.493)	2177	0.287 (0.453)	1 966	0.131***
Father died before 65	0.161 (0.368)	4143	0.158 (0.365)	2177	0.165 (0.371)	1966	− 0.007
Mother died before 65	0.0934 (0.291)	4143	0.0973 (0.297)	2177	0.089 (0.285)	1966	0.008
Start work before 20	0.584 (0.493)	4143	0.537 (0.499)	2177	0.635 (0.481)	1966	− 0.098***
Ever self-employed	0.144 (0.351)	4143	0.194 (0.396)	2177	0.087 (0.283)	1966	0.107***
Unemployment days (59–64)	0.096 (0.489)	4143	0.064 (0.406)	2177	0.132 (0.564)	1966	− 0.067***
Labour income (59–64)	18.53 (11.15)	4143	20.46 (11.88)	2177	16.40 (9.862)	1966	4.06***
Capital income (59–64)	1.446 (10.45)	4143	1.707 (12.52)	2177	1.158 (7.507)	1966	0.549*
Sick leave days (59–64)	0.662 (1.767)	4143	0.489 (1.414)	2177	0.853 (2.074)	1966	− 0.364***

Mean coefficients; standard deviation in parentheses. ***p < 0.01, **p < 0.05, *p < 0.1. Labour income and capital income are measured by 100,000 SEK. Sick leave and unemployment days are measured in 100 days

## Baseline results

To assess the health effect of working after the normal retirement age of 65, we use a linear probability model. Table 3 reports the estimated results.

**Table 3:** The health effect of prolonging working life after the normal retirement age. *Source*: Own calculations, based on register data, LISA database from Statistics Sweden and our survey questionnaire

	(1)	(2)	(3)	(4)	(5)	(6)	(7)	(8)
	Health	Health	Health	Health	Health	Health	Health	Health
Treatment	0.139*** (0.022)	0.109*** (0.023)	0.108*** (0.023)	0.103*** (0.023)	0.079*** (0.023)	0.068*** (0.023)	0.070*** (0.024)	0.103*** (0.039)
Retirement duration								− 0.005 (0.008)
Treatment × retirement duration								− 0.015** (0.008)
Socio-economic variables								
Male		0.002 (0.023)	− 0.000 (0.023)	− 0.002 (0.023)	− 0.042* (0.024)	− 0.041* (0.024)	− 0.037 (0.027)	− 0.043* (0.024)
Immigrant		− 0.116*** (0.039)	− 0.106*** (0.039)	− 0.113*** (0.039)	− 0.097** (0.038)	− 0.095** (0.038)	− 0.093** (0.039)	− 0.092** (0.038)
Age of 68–69		− 0.022 (0.031)	− 0.019 (0.031)	− 0.020 (0.031)	− 0.016 (0.030)	− 0.014 (0.030)	− 0.012 (0.031)	0.002 (0.033)
Age of 70–71		− 0.043 (0.030)	− 0.043 (0.030)	− 0.045 (0.030)	− 0.041 (0.029)	− 0.028 (0.030)	− 0.029 (0.031)	0.004 (0.038)
Age of 72–73		− 0.112*** (0.032)	− 0.111*** (0.032)	− 0.113*** (0.032)	− 0.100*** (0.031)	− 0.089*** (0.032)	− 0.091*** (0.034)	− 0.042 (0.049)
Age of 74–76		− 0.113*** (0.031)	− 0.109*** (0.031)	− 0.109*** (0.031)	− 0.093*** (0.031)	− 0.083*** (0.032)	− 0.089*** (0.033)	− 0.013 (0.061)
Secondary education at 64		0.074*** (0.028)	0.073*** (0.028)	0.066** (0.028)	0.052* (0.028)	0.057** (0.028)	0.056** (0.029)	0.058** (0.028)
Tertiary education at 64		0.213*** (0.030)	0.212*** (0.030)	0.188*** (0.034)	0.143*** (0.035)	0.148*** (0.035)	0.145*** (0.038)	0.149*** (0.035)
Married		0.041 (0.036)	0.042 (0.036)	0.040 (0.036)	0.033 (0.035)	0.033 (0.035)	0.029 (0.036)	0.034 (0.035)
Divorced		0.011 (0.056)	0.011 (0.056)	0.011 (0.056)	0.002 (0.056)	0.002 (0.057)	0.002 (0.058)	0.004 (0.056)
Widowed		0.032 (0.053)	0.033 (0.053)	0.033 (0.053)	0.028 (0.052)	0.032 (0.053)	0.031 (0.054)	0.038 (0.053)
Proxy for life expectancy								
Father died before 65			− 0.079*** (0.028)	− 0.078*** (0.028)	− 0.078*** (0.028)	− 0.074*** (0.028)	− 0.072** (0.029)	− 0.077*** (0.028)
Mother died before 65			− 0.039 (0.038)	− 0.040 (0.037)	− 0.043 (0.037)	− 0.047 (0.036)	− 0.044 (0.036)	− 0.047 (0.036)
Labour market experience								
Started work before 20				− 0.043 (0.026)	− 0.027 (0.026)	− 0.022 (0.026)	− 0.028 (0.027)	− 0.022 (0.026)
Ever self-employed before				0.033 (0.035)	0.087** (0.036)	0.078** (0.035)	0.083** (0.036)	0.067* (0.036)
Unemployment days (59–64)				0.008 (0.020)	0.020 (0.020)	0.017 (0.020)	0.012 (0.020)	0.017 (0.020)
Financial conditions								
Labour income (59–64)					0.007*** (0.001)	0.006*** (0.001)	0.005*** (0.001)	0.006*** (0.001)
Capital income (59–64)					0.001 (0.001)	0.001 (0.001)	0.001 (0.001)	0.001 (0.001)
Previous health								
Sick leave days (59–64)						− 0.024*** (0.005)	− 0.023*** (0.006)	− 0.024*** (0.006)
Industry dummies at 64	No	No	No	No	No	No	Yes	No
Observations	4143	4143	4143	4143	4143	4143	3899	4143
R-squared	0.018	0.060	0.064	0.066	0.085	0.096	0.099	0.098

Robust standard errors in parentheses. ***p < 0.01, **p < 0.05, *p < 0.1. All regressions are adjusted by weights. Labour and capital income are measured in 100,000 SEK. Unemployment and sick leave days are measured in 100 days. The industry dummies are based on the 2002 Swedish industry classification at one-digit level

In the first column of Table 3, we estimate the treatment effect on the propensity of reporting a better health without adding any control variables. The estimated treatment effect is 0.139, suggesting that having work experience after the retirement age of 65 would on average increase the probability of reporting a better health during retirement by about 14%. However, we know that a part of the health difference is explained by observed demographic and socio-economic characteristics, particularly age and educational attainment. Therefore, in column 2, we add such control variables. The estimated coefficient associated with the treatment variable declines to 0.109. Life expectancy may have important influence on both the retirement decision and own health. Therefore, in column 3, we include variables that measure whether the respondent’s father or mother died before 65 as a proxy for own perceived life expectancy. Those two variables may also capture the intergenerational transmission of health. Compared with column 2, including these two variables in column 3 do not significantly change the estimated coefficient associated with the treatment variable. In column 4, we control for a set of variables that relate to previous labour market experience which may affect people’s retirement decision. The estimated coefficient associated with the treatment variable declines to about 0.103. In column 5, we further control for individual financial and income conditions before the normal retirement age of 65 since earlier literature has identified that individual’s financial situation is closely related to the retirement decision and health investment. Controlling for the total labour and capital income between 59 and 64, the estimated treatment effect dramatically drops from 0.103 in column 4 to 0.079 in column 5. It is interesting to point out that it is only the estimated coefficient associated with labour income that is significant while the estimated coefficient on capital income is not statistically significant. One possible explanation is that labour income may also capture other work-related effects, such as a good working environment. As previously mentioned the estimated treatment effect might be upward biased due to healthier people who are more likely to stay longer in the labour market. To capture the selection of healthy people into the treatment group, we further control for the total sick leave days between 59 and 64 years old (column 6). We use this variable as a proxy for individual health conditions before the normal retirement at the age of 65, even though we are aware that our proxy does not address the selection issue perfectly. The estimated effect further decreases from 0.079 in column 5 to 0.068 in column 6 but remains statistically significant. This result indicates that the positive association between the prolonging working life after age of 65 and a better health remains even after we have controlled for the pre-retirement health condition. Working environment may differ among industries, which is an important factor in affecting worker’s health and retirement decision. Therefore, in column 7, we include industry dummies in which people worked at age of 64. The estimated health effect does not change much compared to column 6.

Even though we found a positive health effect of prolonging working life after the normal retirement age of 65, this positive effect of working after 65 may however differ according to the length of retirement. It is reasonable to believe that the health benefit might be stronger for those who have worked and recently retired compared with those who have worked but have been away from the labour market for a longer time. Cohen [13] suggests that social interaction is strongly correlated with physical and mental health through the adjustments of health behaviours due to social influences from peers, information and economic services. Thus, it can be expected that the health benefits of social interactions decline over time and hence negatively related to the length of retirement. Therefore, in column 8, we check whether the health benefits of working after age 65 is transitory or it has long-lasting effect by interacting the treatment variable with the length of retirement. The length of retirement is defined as the time span between the current age and the age at which the individual definitively exited the labour force for the treatment group and as the time span between the current age and age 65 for the control group. The estimated interaction coefficient is about − 0.015 and statistically significant, suggesting the health benefit would disappear after 6 years.^10^

To sum up, our analysis shows that the treatment group has on average about 6.8% higher probability of reporting better health during retirement compared to our control group. In other words, for all retired people, prolonging working life after the normal retirement age of 65 increases the probability of having a better current health by 6.8%. Moreover, we see that the health effect is still significant even after controlling for pre-retirement health, indicating the positive effect is less likely to be entirely driven by selection of healthy people. Furthermore, the effect of extending working life after retirement age has only a transitory positive impact on health. In the medium or long run, we found no health benefit of working after the normal retirement age of 65. A possible explanation is that people adjust their behaviour gradually after retirement. When people have been participating more recently in the labour market, late-life work may transfer into better health through social contacts and interactions. However, such beneficial effects on health disappear as the period of retirement increases.

However, it is important to stress that the sick leave data might not perfectly capture the individual’s pre-retirement health status. The sick leave data only tells about the number of days that people are granted for the sick leave without saying anything about the severity of the illness. Thus, this variable may not fully reflect people’s health condition. Additionally, we are not sure that any other selection on unobservable would drive this result. For instance, individuals who anticipate a potential health gain from prolonging their working after normal retirement age of 65 could have the most incentive to stay in the labour market as long as possible, biasing our results upwards.

## Heterogeneity

### Socio-economic groups

In this section, we do several sub-sample analyses to check if the positive relationship between working after the normal retirement age and health differs among different socio-economic groups. Specifically, we analyse the potential health effect according to gender, marital status, income level, and skill level.

**Table 4:** The health effect of prolonged working life, by socio-economic factors. *Source*: Own calculations, based on register data, LISA database from Statistics Sweden and our survey questionnaire

	(1)	(2)	(3)	(4)	(5)	(6)	(7)	(8)	(9)
	Gender		Marital status		Labour income level		Skill level		
	Male	Female	Married	Single^a^	Low income^b^	High income^c^	Low skill	Medium skill	High skill
Treatment	0.078** (0.031)	0.046 (0.035)	0.077*** (0.029)	0.043 (0.041)	0.107 (0.067)	0.038 (0.045)	0.084 (0.063)	0.080** (0.031)	0.040 (0.041)
Observations	2284	1859	2878	1265	586	1157	439	2375	1276
*R* ^2^	0.099	0.106	0.118	0.077	0.102	0.118	0.207	0.072	0.151

Robust standard errors in parentheses.***p < 0.01, **p < 0.05, *p < 0.1. All regressions are adjusted by weights. The control variables are the same as column 6 in Table 3

^a^Single includes people whose marital status are single, widow and divorced

^b^Low income is defined as total labour income (age 59–64) below 20th percentile of the labour income distribution

^c^High income is defined as total labour income (age 59–64) above 80th percentile of the labour income distribution. Regarding the three skill levels, see variable definition, Table 6 in the appendix

Table 4 reports the results of the separate regressions based on gender, marital status, labour income and skill level. The control variables are the same as in column 6 in Table 3. Regarding gender difference, we found that the positive health effect of working after 65 is restricted to male respondents (see from column 1 and 2 in Table 4). The estimated coefficient for males is about 0.078 and is statistically significant while the estimated coefficient for females is statistically insignificant. Regarding marital status (married vs. single), we found that the positive association between health and prolonging working life after age of 65 only is confined to married people (see column 3 and 4 in Table 4). With regard to income, we define the low-income group as respondents with a total labour income (between age of 59 and 64) falling below the 20th percentile of the labour income distribution. Similarly, we define the high-income group as individuals with a total labour income that is above the 80th percentile. The regression results for the low- and high-income groups are shown in Table 4, column 5 and 6. The estimated coefficients are positive but not statistically significant at conventional level for both income groups. When we restrict the sample to respondents falling between 20th and 80th percentile of labour income distribution (middle-income groups), we found a significant positive health effect.

To further test if our results are similar between different socio-economic groups, we estimate the same model by skill levels. Using the Swedish occupational classification (SSYK), we follow Anxo [1] and divide the sample into three skill groups: low-, medium- and high-skilled workers, where the classification takes into account both the type of occupation and educational attainment.^11^ As shown in Table 4, the beneficial effect of extending working life is limited to medium skilled workers. There are reasons to think that skill levels reflect both working conditions/job content and social/income classes [1]. Our results show therefore that the positive health effect of working beyond standard retirement age is restricted to medium-skilled workers. The results also show that the treatment variable is not statistically significant among the low-skilled and high-skilled sample, which is consistent with the results for income groups (column 5 and 6). This result might be due to a stronger positive correlation between investment in health and human capital for middle-income groups. However, we should emphasise that the number of observations among low-skilled and low-income groups is significantly smaller than for other groups, which could explain why the estimated effect is statistically insignificant.

### The effect of prolonging working life on alternative health-related outcomes

**Fig. 5:**
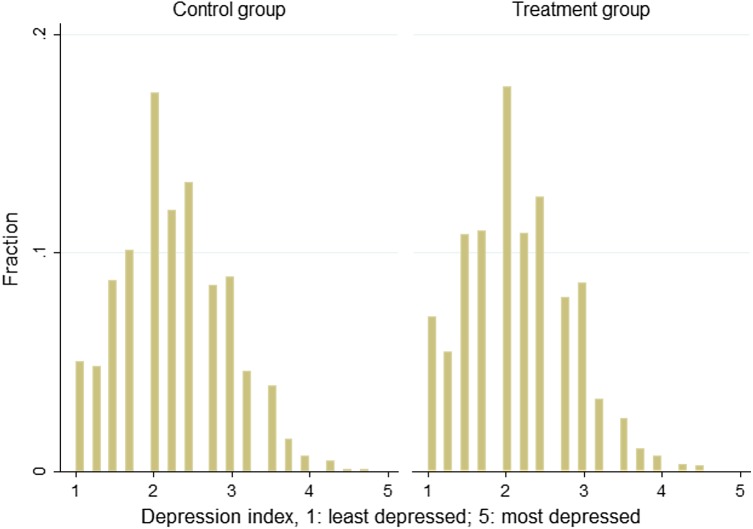
Depression index

**Fig. 6:**
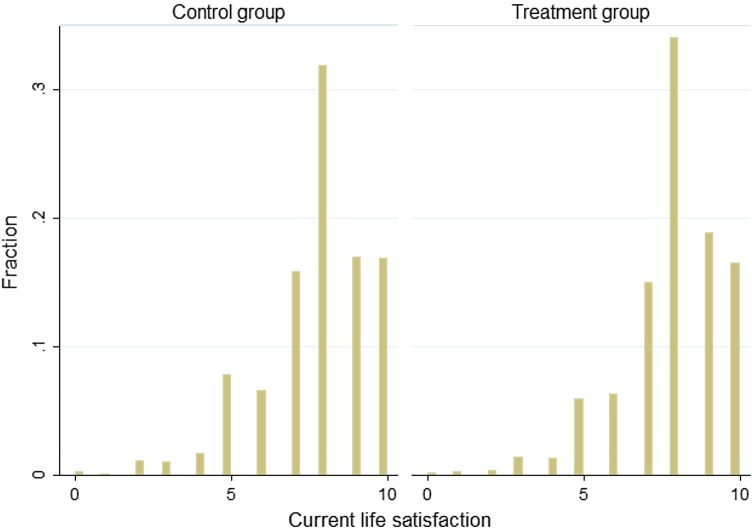
Current life satisfaction

**Fig. 7:**
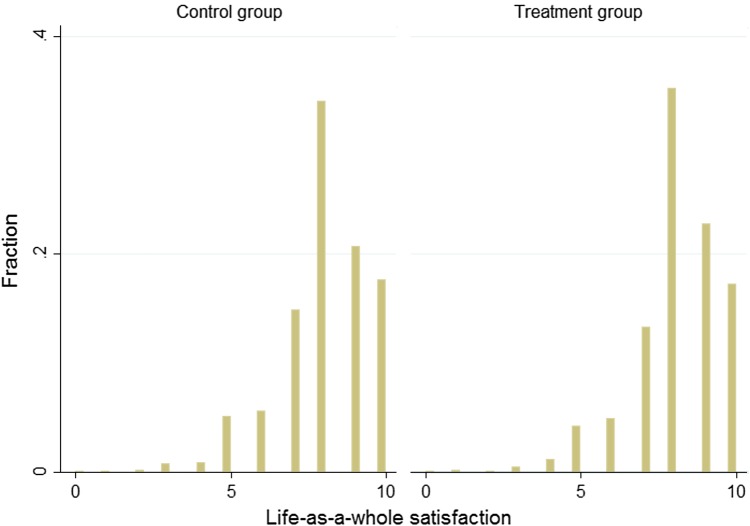
Life as-a-whole life satisfaction

Self-assessed health reflects subjective health conditions. However, it is likely that the effect of late work-life affects overall health positively through more than one specific aspect of health. Therefore, we investigate the treatment effect on alternative health-related measures, specifically physical health/conditions, mental health (to feel depressed), satisfaction with current live and overall life satisfaction. We show the histograms of “depression index”, current life satisfaction and life-as-a-whole life satisfaction for both the treatment and control group in the appendix (Figs. 5, 6, 7).

**Table 5:** The effect of prolonging working life on alternative health-related outcomes, OLS estimations. *Source*: Own calculations, based on register data, LISA database from Statistics Sweden and our survey questionnaire

	(1)	(2)	(3)	(4)
	Physical condition	“Depression” index	Current life satisfaction	Life-as-a-whole life satisfaction
Treatment	0.011 (0.023)	− 0.029 (0.034)	0.058 (0.082)	0.032 (0.070)
Observations	4143	3901	3998	4038
*R* ^2^	0.100	0.066	0.060	0.053

Robust standard errors in parentheses. ***p < 0.01, **p < 0.05, *p < 0.1. All regressions are adjusted by weights. The control variables are the same as column 6 in Table 3

Table 5 displays the OLS regression results for the effect of prolonging working life after 65 on alternative health-related outcomes. These variables are related to specific health outcomes and may therefore help us to understand through which channels an extended working life may improve health. As shown by column 1, we find that the treatment group is more likely to experience a deterioration in physical fitness. However, the estimated effect is statistically insignificant, indicating that working after 65 does not significantly affect physical fitness during retirement. We also found a negative correlation between the treatment variable and our “depression index” but the effect is also in this case not statistically significant (see column 2). The level of current life satisfaction and life-as-a-whole life satisfaction are not statistically different between our treatment and control groups (see column 3 and 4). Overall, we do not find any evidence that prolonging working life after 65 would have any positive health effects due to differences in physical fitness, depressive tendencies or well-being.

## Conclusion

The results of our estimations suggest that working after standard retirement age has a weak but positive effect on subjective health. However, even though we may reject the hypothesis that a prolonged working life worsens health, our results show that the positive impact of extending working life is small, transitory and disappears after a period of 6 years. One plausible explanation for this is that the “good” health effects of working longer related to, for example, social interactions, disappear as soon as individuals are no longer exposed to the positive impact of work environment.

A previous study by Hagen [17] found no effects on mortality and healthcare utilization when the entitlement to retire with full pension benefits was raised from 63 to 65 among Swedish female local government workers. In contrast to Hagen [17] our study relies on a representative sample of older individuals aged 66–76. However, our results are consistent with his findings as the positive health effects of working after 65 seem to be temporary.

In spite of the fact that we control for individual’s health status prior to normal retirement age, the reader should be aware that we cannot control for all unobserved factors which may affect both health and the decision to stay in the labour market after 65. The reader should refrain therefore from interpreting our results as causal, i.e., that delaying the exit from the labour force improves individuals’ health during the early phase of retirement. In other words, we cannot exclude that we have still a situation of unobserved heterogeneity and omitted variable bias.

To test the robustness of our estimations we used alternative econometric methods and found that our results are quite stable independently of the estimation methods used. Furthermore, separate regressions reveal that the positive health effects of working after 65 are limited to male, married, medium-skilled workers and to the middle-income group. Using alternative dependent variables such as physical fitness, mental health (tendency to be depressed) or life satisfaction, we found no statistically significant differences between our control and treatment groups. A plausible explanation is that senior work implies social and psychological stimulation improving subjective health, while well-being, depressive tendencies or physical capabilities are dependent on other factors and are not affected by a late exit from the labour force. In other words, the positive impact we found on subjective health cannot be ascribed to these other health dimensions. In this paper, we only consider self-assessed health and physical fitness without considering health in terms of overall mental well-being and/or cognitive capacity. It would be therefore interesting for future work to extend the research to encompass these other dimensions of health and well-being.

## Appendix

See Figures 4, 5, 6 and 7 and Tables 6, 7, and 8.

**Table 6:** Variable description

Variables	Definition
Dependent variables	
Self-assessed current health	1–5, 1 = very poor; 2 = poor; 3 = fair; 4 = very good; 5 = excellent, survey questionnaire
Health (current)	= 1 if current health is excellent or very good, zero otherwise, survey questionnaire
Physical condition	= 1 if deterioration of physical conditions, zero otherwise, survey questionnaire
Depression index	Scale 1–5, 1 = least depressed, 5 = very depressed, survey questionnaire
Current life satisfaction	Scale 1 to 10, 1 least satisfied 10 most satisfied, survey questionnaire
Life-as-a-whole life satisfaction	Scale 1 to 10, 1 least satisfied 10 most satisfied, survey questionnaire
Explanatory variables	
Male	Male, dummy variable, LISA
Age 66–67	Age between 66 and 67 at the survey time, dummy variable, LISA
Age 68–69	Age between 68 and 69 at the survey time, dummy variable, LISA
Age 70–71	Age between 70 and 71 at the survey time, dummy variable, LISA
Age 72–73	Age between 72 and 73 at the survey time, dummy variable, LISA
Age 74–76	Age between 74 and 76 at the survey time, dummy variable, LISA
Foreign born	Born outside Sweden, dummy variable, LISA
Elementary school at 64	Compulsory school or less, dummy variable, LISA
Secondary education at 64	Secondary education, dummy variable, LISA
Tertiary education at 64	University education, dummy variable, LISA
Single	Current civil status, single, dummy variable, Questionnaire
Married	Current civil status, married/cohabiting, dummy variable, Questionnaire
Divorced	Current civil status, divorced, dummy variable, Questionnaire
Widowed	Current civil status, widow/widower, dummy variable, Questionnaire
Age started working	Age the respondent started to work, years, Questionnaire
Started working before 20	Dummy variable, based on the previous variable, Questionnaire
Father death before 65	Age of death of the father younger than 65, dummy variable, Questionnaire
Mother death before 65	Age of death of the mother younger than 65, dummy variable, Questionnaire
Ever self-employed	Ever self-employed upto or at 64 years old, LISA
Labour income	Labour income between 59 and 64 years, in 100,000 SEK, LISA
Capital income	Capital income between 59 and 64 years, in 100,000 SEK, LISA
Sick leave days	Number of sick days (in 100 days), between 59 and 64 years, LISA
Unemployment days	Unemployment spell in days (in 100 days), between 59 and 64 years, LISA
Agriculture	Worked in agriculture dummy variable, LISA, (2002 Swedish industry classification)
Manufacturing	Worked in manufacturing, dummy variable, LISA
Energy	Worked in energy sector, dummy variable, LISA
Construction	Worked in construction, dummy variable, LISA
Trade and communication	Worked in trade and communication, dummy variable, LISA
Finance and business service	Worked in finance and business, dummy variable, LISA
Public administration	Worked in public administration, dummy variable, LISA
Education and research	Worked in education and research, dummy variable, LISA
Health and social care	Worked in health sector, dummy variable, LISA
Culture and personal service	Worked in culture and personal service, dummy variable, LISA
Unknown sector	Worked in unknown sector, dummy variable, LISA
Classification of skill groups	
High skill	Worked in occupations as legislators, senior officials and managers, professionals. Dummy variable, LISA
Medium skill	Worked in occupations as technicians and associate professionals, teaching professionals, service workers and shop and market sales workers, clerks, plant and machine operators and assemblers. Dummy variable, LISA
Low skill	Worked in occupations as skilled agricultural and fishery workers, craft and related trade workers, elementary occupations. Dummy variable, LISA

*LISA* Longitudinal Integration Database for Health Insurance and Labour Market Studies, Statistics Sweden

**Table 7:** The health effect of prolonging working life, by retirement length. *Source*: Own calculations, based on register data, LISA database from Statistics Sweden (SCB 2016) and our survey questionnaire

	(1)	(2)	(3)	(4)
	0–2 years	3–5 years	6–8 years	9–11 years
Treatment	0.116*** (0.031)	0.076 (0.066)	− 0.000 (0.077)	− 0.038 (0.099)
Observations	2495	778	562	308
*R*-squared	0.105	0.12	0.112	0.103

Robust standard errors in parentheses. ****p* < 0.01, ***p* < 0.05, **p* < 0.1. All the regressions are adjusted by weights. All control variables are the same as column 6 Table 3

**Table 8:** The health effect of prolonging working life after the normal retirement age of 65, studied using alternative estimation methods

	(1)	(2)	(3)
	Probit model^a^	OLS^b^	Ordered probit model^c^
Treatment	0.064*** (0.022)	0.145*** (0.039)	0.197*** (0.052)
Observations	4143	4143	4143
*R* ^2^	0.076	0.097	0.042

Robust standard errors in parentheses. ****p* < 0.01, ***p* < 0.05, **p* < 0.1. All regressions are adjusted by weights. The control variables are the same as column 6 in Table 3

^a^Column 1 shows the estimated marginal effect from the probit model. The outcome variable is the binary health variable

^b^The outcome variable in column 2 is a self-assessed health variable that is measured by a 5-scale score

^c^Column 3 shows the estimated coefficients from the ordered probit model, where the self-assessed health variable is measured by a 5-scale score

## Footnotes

1 According to the Labour force surveys by Statistics Sweden [26] the employment rate among Swedish workers aged 55–64 was in 2016 one of the highest among modern economies, namely 77.6% for Swedish men and 73.5% for Swedish women. At the same date, Sweden exhibited also the highest employment rate among senior workers (65–74) in the EU, 19.2 for men and 12.5% for women.

2 In Sweden, all residents who have income from work, unemployment benefits or student benefits are eligible and covered by sickness insurance.

3 A description of LISA database can be found at Statistics Sweden home page, https://www.scb.se/en/services/guidance-for-researchers-and-universities/vilka-mikrodata-finns/longitudinella-register/longitudinal-integration-database-for-health-insurance-and-labour-market-studies-lisa/.

4 The Swedish versions of the two questionnaires are available upon request.

5 We exclude also bridge workers returning to the labour market after being fully retired, i.e. about 300 observations.

6 The four questions are: (1) Do you feel often alone and depressed? (2) Do you feel sometime worthless? (3) Do you feel often sad and distressed? (4) Do you feel often helpless and would like someone help you to solve your problems?

7 We have performed a robustness analysis in order to assess the impact of dichotomisation: our results are robust and our main conclusions not affected by the way we dichotomize the health variable.

8 In a previous report [2], we analysed, using standard econometric techniques (multinomial logit), the extent to which socio-economic factors and individual characteristics influence senior citizens’ decisions to retire or to stay longer on the labour market. The results of our estimations show clearly that good health, high educational attainment, good working conditions and employment status (to be self-employed) are strong predictors of working beyond 65-year-old. We found also that job quality and work satisfaction are essential elements for motivating seniors to postpone retirement. Family norms regarding retirement as well as perceived life expectancy are also important factors affecting the decision to stay or not on the labour market after 65 years old. We found also that respondents belonging to younger cohorts are also more prone to continue working after 65. A possible explanation is that younger cohorts are to a larger extent than the older one exposed to the new pension system that creates strong economic incentives to postpone the retirement decision and work longer.

9 Ideally, we wanted to control for family norm by using the information whether the respondent’s father or mother retired before 65. However, the number of observations dropped dramatically. Therefore, we decided to exclude these two variables. However, our results are robust when we include these variables.

10 We also estimated separate equations according to the length of retirement. Again, we found that the health benefits are only transitory (see Table 7 in the appendix). Furthermore, we performed a robustness analysis using non-linear models and the results are consistent with our baseline findings (see Table 8 in appendix).

11 See Table 6 in appendix for a precise definition of the three skill groups.
